# Asymmetry of Hemispheric Network Topology Reveals Dissociable Processes between Functional and Structural Brain Connectome in Community-Living Elders

**DOI:** 10.3389/fnagi.2017.00361

**Published:** 2017-11-03

**Authors:** Yu Sun, Junhua Li, John Suckling, Lei Feng

**Affiliations:** ^1^Centre for Life Science, Singapore Institute for Neurotechnology (SINAPSE), National University of Singapore, Singapore, Singapore; ^2^Brain Mapping Unit, Department of Psychiatry, University of Cambridge, Herchel Smith for Brain and Mind Sciences, Cambridge, United Kingdom; ^3^Department of Psychological Medicine, Yong Loo Lin School of Medicine, National University of Singapore, Singapore, Singapore

**Keywords:** resting-state fMRI, diffusion tensor imaging (DTI), graph theory, hemispheric asymmetry, brain networks

## Abstract

Human brain is structurally and functionally asymmetrical and the asymmetries of brain phenotypes have been shown to change in normal aging. Recent advances in graph theoretical analysis have showed topological lateralization between hemispheric networks in the human brain throughout the lifespan. Nevertheless, apparent discrepancies of hemispheric asymmetry were reported between the structural and functional brain networks, indicating the potentially complex asymmetry patterns between structural and functional networks in aging population. In this study, using multimodal neuroimaging (resting-state fMRI and structural diffusion tensor imaging), we investigated the characteristics of hemispheric network topology in 76 (male/female = 15/61, age = 70.08 ± 5.30 years) community-dwelling older adults. Hemispheric functional and structural brain networks were obtained for each participant. Graph theoretical approaches were then employed to estimate the hemispheric topological properties. We found that the optimal small-world properties were preserved in both structural and functional hemispheric networks in older adults. Moreover, a leftward asymmetry in both global and local levels were observed in structural brain networks in comparison with a symmetric pattern in functional brain network, suggesting a dissociable process of hemispheric asymmetry between structural and functional connectome in healthy older adults. Finally, the scores of hemispheric asymmetry in both structural and functional networks were associated with behavioral performance in various cognitive domains. Taken together, these findings provide new insights into the lateralized nature of multimodal brain connectivity, highlight the potentially complex relationship between structural and functional brain network alterations, and augment our understanding of asymmetric structural and functional specializations in normal aging.

## Introduction

The human brain is structurally and functionally asymmetrical or lateralized (Galaburda et al., 1978; Toga and Thompson, 2003). Particularly, a number of brain phenotypes have been shown to exhibit asymmetry, including gray matter volume (Good et al., 2001; Lancaster et al., 2003; Takao et al., 2011a), cortical thickness (Zhou et al., 2013), and white matter integrity (Cao et al., 2003; Takao et al., 2011b; Thiebaut de Schotten et al., 2011b; Song et al., 2014), which show varying degrees of correspondence to brain functions (Toga and Thompson, 2003; Herve et al., 2013) and that this asymmetry is hormone related (Hausmann and Gunturkun, 2000; Hausmann et al., 2003). For instance, accumulating evidences have revealed a prominent brain asymmetry—the so-called Yakovlevian torque, which demonstrates the right frontal and left occipital petalias, in the development of asymmetry (Toga and Thompson, 2003). Furthermore, leftward volume asymmetries have been consistently observed in the inferior frontal gyrus and the superior temporal gyrus, which are believed as an anatomical marker of left hemispheric functional specialization for language processing (Geschwind and Levitsky, 1968). While rightward asymmetry of gray matter volume in the lateral inferior frontal gyrus and diffusion parameters of frontal white matter tracts have also been frequently reported (Zhou et al., 2013), indicating a rightward predilection to processing non-verbal functions, including spatial attention, and visuospatial memory (Thiebaut de Schotten et al., 2011a). Moreover, studies have reported that brain asymmetries would be altered due to behavior changes in maturation/normal aging (Cabeza, 2002; Zhong et al., 2016), and in various neuropsychiatry (e.g., schizophrenia) as well as neurological (e.g., dementia) diseases (Crow et al., 1989; Thompson et al., 2003; Kim et al., 2012; Wachinger et al., 2016; Sun et al., 2017).

Notably, age-related thinning of the cortical mantle varies regionally, which leads to continuous structural and functional changes of hemispheric asymmetry throughout the lifespan (Zhou et al., 2013). For instance, convergent evidence showed that the asymmetry of regional gray matter volume that is present at birth undergoes a developmental progression in childhood and adolescence as a result of bilateral cortical maturation (Giedd et al., 1996, 1999; Reiss et al., 1996). In older adults, however, a hemispheric asymmetry reduction has been repeatedly revealed in functional neuroimaging studies (Casey et al., 2005; Colcombe et al., 2005; Zuo et al., 2010). Particularly, Cabeza introduced a cognitive neuroscience model, named HAROLD (hemispheric asymmetry reduction in older adults), which is believed to be associated with a functional compensation against aging (Cabeza, 2002). Furthermore, a recent longitudinal study of cortical thickness in normal aging revealed a general thinning in the left hemisphere in contrast to a localized thinning mainly in the parietal regions of the right counterpart (Thambisetty et al., 2010). Of note, the reported aberrations of hemispheric asymmetry in normal aging are examined exclusively at a region level. Until recently, lateralized characteristics of hemispheric brain networks were beginning to be revealed (Iturria-Medina et al., 2011; Tian et al., 2011; Ratnarajah et al., 2013; Caeyenberghs and Leemans, 2014; Zhong et al., 2016). For instance, Iturria-Medina et al., employed DTI tractography (a technique to reconstruct white matter fiber pathways) to investigate the differences in network architecture between the hemispheres in healthy right-handed adults and found that the right hemisphere is more efficient and interconnected in comparison with a more regional central/indispensable architecture in the left hemisphere (Iturria-Medina et al., 2011). Similar hemispheric lateralization in structural brain networks was also observed in Zhong et al. (2016). Using similar graphic analysis framework, Tian investigated the hemispheric topology of functional brain networks in healthy right-handed adults and revealed no significant lateralization (Tian et al., 2011), reiterating the complex hemispheric asymmetry patterns between structural and functional brain networks. Despite these recent advances in brain asymmetry research, however, our understanding about the topological organization of functional and structural hemispheric brain network in aging populations is still rudimentary (Yang et al., 2017).

As illustrated by several of the aforementioned studies, network analysis is an ideal method for obtaining summary measures of cortical connectivity to compare hemispheric topological characteristics. This method allows the measurement of both the strength of local networks via clustering, as well as global network integrity via measures of path length (Jahanshad et al., 2013; Shu et al., 2015). With this in mind, we employed connectomic techniques on resting-state functional as well as diffusion imaging data in a sample of healthy older adults. A graph theory analysis framework was then utilized to investigate the hemispheric brain network topology in these healthy aging adults. Given that converging evidence shows small-world characteristic (as having high local clustering and short paths between brain regions) in hemispheric networks (Iturria-Medina et al., 2011; Tian et al., 2011; Zhong et al., 2016; Sun et al., 2017) and impaired structural/functional connectivity in aging adults (Ferreira and Busatto, 2013; Zuo et al., 2017), we hypothesized that: (1) although the optimal small-world topology would be preserved in the hemispheric networks, different hemispheric asymmetry patterns would be found between functional and structural brain networks; (2) there would be an association between the asymmetry scores and behavioral performance of cognitive functions at various domains.

## Methods and materials

### Subjects

Seventy-six community-dwelling older adults [age = 70.08 ± 5.30 years (mean ± *S.D*.), ranged 60–82 years, male/female = 15/61] were recruited in the western region of Singapore. All subjects were right-handed according to the Modified Edinburgh Questionnaire (Schachter et al., 1987). All participants were pre-screened to ensure that they met all inclusion criteria in the present study; i.e., participants reporting terminal illness, or any contraindication to MRI, or participants who obtained a Clinical Dementia Rating (CDR) global score greater than zero, or participants with any psychiatric or psychological problems were excluded for the current study. Assessments of cognitive ability administered by trained raters included the mini-mental state examination (MMSE) (Folstein et al., 1975; Feng et al., 2012), the Montreal cognitive assessment (MoCA) (Nasreddine et al., 2005; Liew et al., 2015), the Rey auditory verbal learning test (Schmidt, 1996), the Digit Span and Block Design tests from the Wechsler Adults Intelligence Scale (WAIS-III), the Boston Naming Test, the Color Trials Test (CTT), and the Symbol Digit Modalities Test (SDMT). Here are brief explanations of the adopted neuropsychological tests:

- *Rey auditory verbal learning test (RAVLT)*: RAVLT test is employed here to assess verbal memory. During the test, the participant was requested to read a semantically unrelated word list (list A) with 15 words and to recall as many words from the list as possible (immediate recall, referred to hereafter RAVLT_ir_). After five trials of immediate recall, a second interference list (list B) was presented in the same manner. After a 30 min delay, participants were asked to recall the words from list A (delayed recall, referred to hereafter RAVLT_dr_).
- *Digit span*: after the examiner reads a sequence of numerical digits, participants were requested to recall the string correctly (forward, referred to hereafter DigitSpan_fwd_). The length of the digit sequence was increasing in each trial. In the backward condition (referred to hereafter DigitSpan_bwd_), subjects were asked to recall the sequence in reverse order. The longest number of sequential digits that could be corrected recalled was considered as the participant's span.
- *Block design*: the participant is requested to replicate models or pictures of two-color designs using blocks. Difficulty of block design was manipulated with block numbers, e.g., from two-block design to nine-block design in the current work.
- *Color trails test (CTT)*: The test uses numbered colored circles and universal sign language symbols. For the Color Trails 1 trial, the examinee uses a pencil to rapidly connect circles numbered 1 through 25 in sequence. For the Color Trials 2 trial, the examinee rapidly connects numbered circles in sequence, but alternates between pink and yellow colors (Feng, 2017).
- *Boston naming test*: the examinee is requested to tell the examiner the name of each of a series of pictures. The examiner writes down the subject's responses in detail using codes.
- *Symbol digit modality test (SDMT)*: in the written version, the examinee is asked to write as many numbers as he/she can in the boxes below a series of symbols according to the key provided at the top of the page within 90 s. In the oral version, the examiner records the numbers spoken by the subjects.

The neuropsychological tests were conducted from Sep. 2015 to Oct. 2015 in a quite room at our study center, the Training and Research Academy at Jurong Point, Singapore, and the time between testing and the neuroimaging process were 73.0 ± 26.3 days. Detailed demographic and neuropsychological characteristics of the participants are shown in Table 1. The Institutional Review Board of the National University of Singapore approved the study protocol as part of baseline assessments under the Choral Singing for Dementia Prevention Trial and written informed consent was obtained from all participants.

**Table 1:** Demographics and neuropsychological features of the samples.

**Characteristics**	**Mean ± *SD***	**Range (Min–Max)**
Gender (male/female)	15/61	
Age	70.08 ± 5.30	60–82
Years of Education	6.00 ± 3.98	0–15
**PSYCHOLOGICAL MEASURES**		
RAVLT_ir_	47.18 ± 10.76	23–71
RAVLT_dr_	10.20 ± 3.04	0–15
DigitSpan_fwd_	10.54 ± 2.65	5–16
DigitSpan_bwd_	6.08 ± 2.21	2–14
SDMT_written_	31.95 ± 11.56	6–54
SDMT_oral_	39.17 ± 12.89	9–67
BostonNaming	22.13 ± 5.08	10–30
BlockDesign	26.93 ± 9.15	3–49
CTT1	69.34 ± 26.86	33–184
CTT2	136.10 ± 43.56	63–270
MMSE	28.25 ± 1.81	22–30
MoCA	25.75 ± 3.48	17–30

*RAVLT_ir_, Rey auditory verbal learning test, immediate recall; RAVLT_dr_, Rey auditory verbal learning test, delayed recall; SDMT, symbol digit modalities test; CTT, color trials test; MMSE, mini-mental state examination; MoCA, Montreal cognitive assessment*.

### Data acquisition

Data acquisition was performed on a 3-T Siemens Prisma scanner (Siemens, Erlangen, Germany) at the Clinical Imaging Research Center (CIRC), National University of Singapore, Singapore. Participants were instructed to keep still and remain as motionless as possible before the scanning. During the data acquisition, no participants fell asleep which was confirmed by self-reports after scanning.

One structural T1-weighted MRI, one resting-state fMRI scanning, and two volumes of diffusion-encoded images were recorded in a single session. Specifically, structural MRI for co-registration and normalization were acquired using a high-resolution T1-weighted magnetization prepared rapid gradient-recalled sequence with the following parameters (TR = 2,300 ms; TE = 2.03 ms; field of view [FOV] = 256 × 256 mm^2^; slice number = 176; acquisition matrix = 256 × 256; voxel resolution = 1 × 1 × 1 mm^3^). Resting-state fMRI data were obtained using a single-shot echo-planar imaging (EPI) sequence of 210 images and the acquisition parameters consisted of the following (TR = 2,550 ms; TE = 30 ms; FOV = 192 × 192 mm^2^; slice number = 42, slice thickness = 3 mm; acquisition matrix = 64 × 64; voxel resolution = 3 × 3 × 3 mm^3^). A single-shot echo-planar sequence (TR = 8,500 ms; TE = 96 ms, FOV = 192 × 192 mm^2^; b-factor = [350 650 1,000 1,300 1,600] s/mm^2^; 1 baseline image with b0 = 0 s/mm^2^) from 12 separate non-parallel directions was utilized to obtain diffusion-encoded images (slide number = 63, slice thickness = 2.0 mm with no gap; acquisition matrix = 96 × 96; voxel resolution = 2 × 2 × 2 mm^3^). The diffusion sequences were scanned twice for better signal-to-noise ratio.

### Functional data preprocessing and network construction

Functional data preprocessing was performed using the Statistical Parametric Mapping (SPM12, http://www.fil.ion.ucl.ac.uk/spm/software/spm12/), resting-sate fMRI data analysis toolkit (Song et al., 2011), and DPARSF (Yan and Zang, 2010). Due to instability of the initial signals, the first 10 volumes were removed for the following analysis. The remaining fMRI images were then corrected for time offsets between slices. The time series of images were then realigned to the first volume to correct the inter-scan head motion using a six-parameter rigid-body transform. The individual anatomical T1-weighted images were coregistered to functional images after motion correction using a linear transformation and were segmented into gray mater, white matter, and cerebrospinal fluid (CSF) tissue maps according to DARTEL (Ashburner, 2007). To reduce the variance estimates, nuisance signal correction was applied on 24 head-motion profiles, white matter, CSF, and global signals. Subsequently, a standard template (Montreal Neurological Institute, MNI) was employed to normalize the resulting motion-corrected volumes, which were further resampled to a 3-mm isotropic resolution and spatially smoothed with an isotropic Gaussian kernel (FWHM = 4.5 mm). Previous studies showed that correlated endogenous dynamics in resting-state functional data are particularly salient in frequencies below 0.1 Hz (Lowe et al., 1998). Therefore, the resulting images were further band-pass filtered (0.01–0.1 Hz) to minimize the effect of very low frequency drift and high frequency physiological noise.

**Figure 1:**
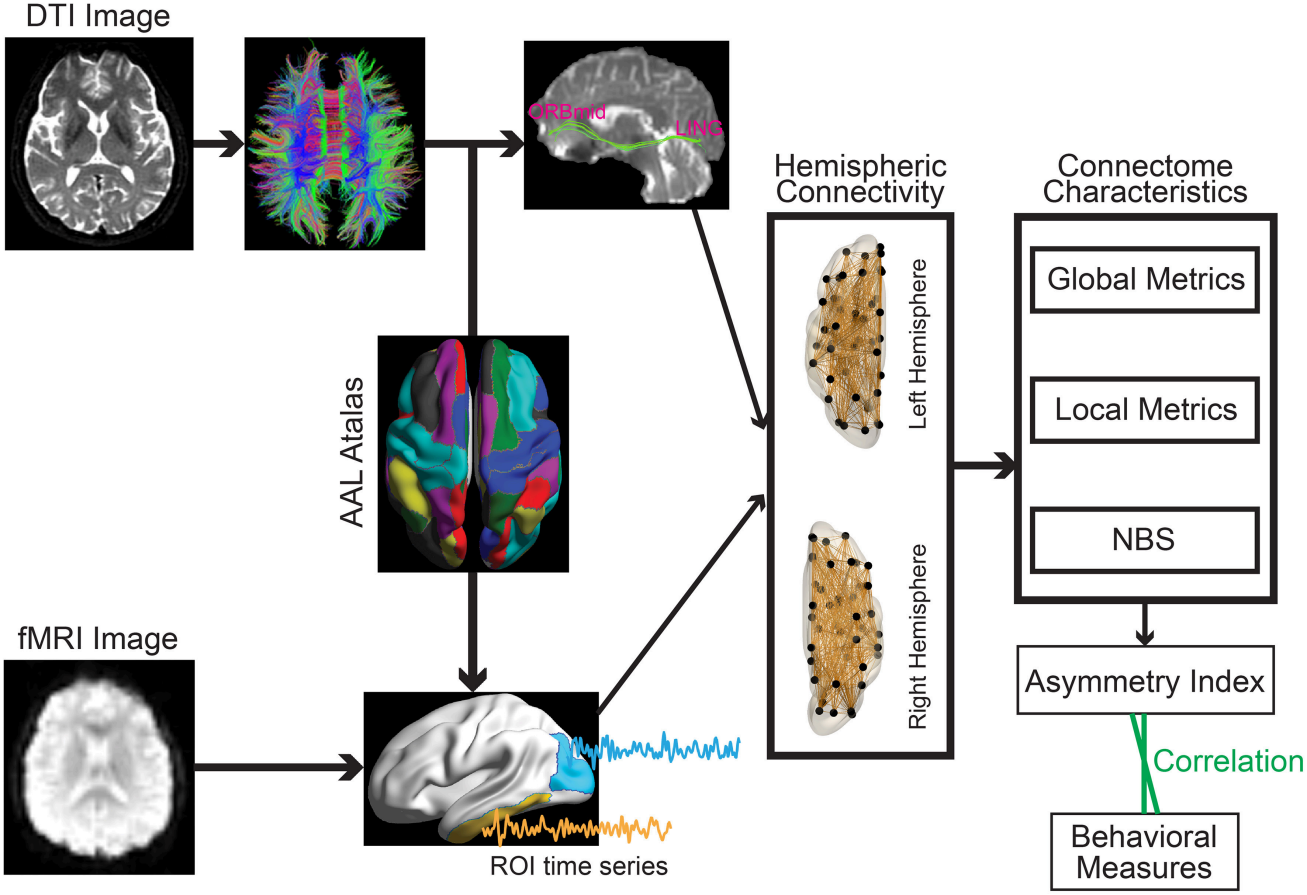
Schematic overview of the formation of the individual hemispheric network for structural **(Upper)** and functional **(Lower)** data.

To define the network nodes, a previously validated and widely used automatically labeled template (AAL-90) was employed in the current work to enable direct comparison with the existing studies and reduce the potential confounding effect during a template-to-template mapping between discordant atlas. Specifically, AAL atlas parcellated the brain into 90 regions of interests (ROIs) with 45 regions in each hemisphere (Table 2) (Tzourio-Mazoyer et al., 2002). A representative time series from each ROI was obtained by averaging the time series of each voxel within that region. Functional connectivity, which examines interregional correlations in neuronal variability, was then obtained through Pearson correlation between any possible pairs of ROIs (Figure 1). Fisher's r-to-z transformation was further applied to the obtained correlation matrices to improve the normality of the correlation coefficients. Given the ongoing debate about the physiological meaning of negative correlation (Chang and Glover, 2009; Anderson et al., 2011), only positive connections were retained.

**Table 2:** The names and corresponding abbreviations of the regions of interest.

**Region name**	**Abbr**.	**Class**
Amygdala	AMYG	Paralimbic
Angular gyrus	ANG	Association
Anterior cingulate gyrus	ACG	Paralimbic
Calcarine fissure	CAL	Primary
Caudate nucleus	CAU	Subcortical
Cuneus	CUN	Association
Fusiform gyrus	FFG	Association
Gyrus rectus	REC	Paralimbic
Heschl gyrus	HES	Primary
Hippocampus	HIP	Subcortical
Inferior frontal gyrus (opercula)	IFGoperc	Association
Inferior frontal gyrus (triangular)	IFGtriang	Association
Inferior occipital gyrus	IOG	Association
Inferior parietal lobule	IPL	Association
Inferior temporal gyrus	ITG	Association
Insula	INS	Paralimbic
Lingual gyrus	LING	Association
Middle cingulate gyri	MCG	Paralimbic
Middle frontal gyrus	MFG	Association
Middle occipital gyrus	MOG	Association
Middle temporal gyrus	MTG	Association
Olfactory	OLF	Paralimbic
Orbitofrontal cortex (superior)	ORBsup	Paralimbic
Orbitofrontal gyrus (inferior)	ORBinf	Paralimbic
Orbitofrontal gyrus (medial)	ORBmed	Paralimbic
Orbitofrontal gyrus (middle)	ORBmid	Paralimbic
Pallidium	PAL	Subcortical
Paracentral lobule	PCL	Association
Parahippocampal gyrus	PHG	Paralimbic
Postcentral gyrus	PoCG	Primary
Posterior cingulate gyrus	PCG	Paralimbic
Precentral gyrus	PreCG	Primary
Precuneus	PCUN	Association
Putamen	PUT	Subcortical
Rolandic operculum	ROL	Association
Superior frontal gyrus (dorsal)	SFGdor	Association
Superior frontal gyrus (medial)	SFGmed	Association
Superior occipital gyrus	SOG	Association
Superior parietal gyrus	SPG	Association
Superior temporal gyrus	STG	Association
Supplementary motor area	SMA	Association
Supramarginal gyrus	SMG	Association
Temporal pole (middle)	TPOmid	Paralimbic
Temporal pole (superior)	TPOsup	Paralimbic
Thalamus	THA	Subcortical

It is well-known that head motion can introduce substantial changes in the time series of resting-state functional connectivity (Power et al., 2012; Van Dijk et al., 2012; Yan et al., 2013). Here, two strategies were adopted in the current study to control for head motion. First, to account for the transient excessive movement, subjects were excluded for further analysis if their head moved more than 2 mm or 2°. Additionally, we addressed the residual effects of head motion through frame-wise displacement (FD) derived with Jenkinson's relative root mean square algorithm (Jenkinson et al., 2002) as nuisance covariate. Subjects with mean FD higher than 1 mm were discarded. Head motion was quite small in the current study and no subjects were removed based upon these two criteria.

### Structural data preprocessing and network construction

Structural data preprocessing and brain network construction were conducted using the FMRIB Software Library (FSL, v5.0, Smith et al., 2004), diffusion toolkit (Wang et al., 2007), and PANDA (Cui et al., 2013) and has been described in detail previously (Sun et al., 2016a, 2017). Here we provide a brief description about the preprocessing steps.

The distortion of diffusion-weighted images was corrected for effects of head motion and eddy currents using an affine alignment of each image to the b0 image. After this process, the six independent components of the diffusion tensor were estimated within each voxel. A widely used deterministic streamline tracking algorithm was then performed to obtain the whole-brain tractography (Mori et al., 1999). The tracking procedure started from the deep white matter regions and terminated if it turned an angle > 45° or reached a voxel with a fractional anisotropy < 0.15. For each participant, the structural brain network was constructed through combining the parcellation map with the white matter tractography (Figure 1). Of note, the individual-based parcellation template that was obtained through weaving the standard AAL template from the MNI space to the DTI native space was employed to define the network nodes. Edge weights were computed as the streamline density (computed as the ratio between the number of streamlines and sum of volumes of the two interconnected ROIs at individual native space) to account for different sizes of the ROIs (Buchanan et al., 2014).

### Graph theory analysis

After the network construction, each individual has one functional brain network and one structural brain network at the whole brain level (90 × 90). In order to assess the topology of multimodal hemispheric networks, we eliminated the interhemispheric connections and only kept intrahemispheric connections (45 × 45) in functional and structural networks for both hemispheres.

Graph theory is a natural framework for the mathematical representation of complex networks, proving a powerful and quantitative way to describe the segregation and integration of the brain network form the perspective of its topological architecture (Sporns, 2011). In this work, we calculated the small-world parameters (including weighted clustering coefficient, *C*_*w*_, weighted characteristic path length, *L*_*w*_, small-worldness, σ, global efficiency, *E*_*global*_, and local efficiency *E*_*local*_) for hemispheric brain networks using the Brain Connectivity Toolbox (Rubinov and Sporns, 2010). *C*_*w*_, *L*_*w*_, and σ were originally introduced in Watts and Strogatz (1998) for quantitatively assessing the small-world properties (high local clustering and short paths between brain regions), whereas *E*_*global*_ and *E*_*local*_ were employed here to provide comprehensive understanding of small-world architecture in terms of information flow (Latora and Marchiori, 2001; Achard and Bullmore, 2007). Nodal efficiency (*E*_*nodal*_) (Achard and Bullmore, 2007), which measures the ability of regional information transmission, was utilized to assess regional properties. We provided the definitions and formulations of the network metrics used in Table 3. More detailed description and usage of the graph theory parameters can be found in Boccaletti et al. (2006), Bullmore and Sporns (2009), and Rubinov and Sporns (2010).

**Table 3:** Formulations and description of topological measurements applied in the current work.

**Network properties**	**Definitions**	**Measurement and meaning**
**GLOBAL PROPERTIES**		
Clustering coefficient (*C_*w*_*)	$C_{w}=\frac{1}{N}\underset{i\in N}{\sum }\frac{\sum_{j,k}{\left(w_{ij}w_{jk}w_{ki}\right)}^{1/3}}{\left(k_{i}\left(k_{i}-1\right)\right)}$	*C_*w*_* measures the extent of a local clustering or cliquishness of a network *G* with *N* nodes. Here *k_*i*_* is the number of edges connecting to node *i, w_*ij*_* is the edge weight between region *i* and *j*.
Characteristic path length (*L_*w*_*)	$L_{w}=\frac{1}{N\left(N-1\right)}\underset{i\in N}{\sum }\underset{i\neq j\in N}{\sum }\min\left\{L_{ij}\right\}$	*L_*w*_* measures the overall routing efficiency of the network. min{*L*_*ij*_}is the shortest path length between node *i* and *j*. Path length of an edge conceptualized to weight graph is defined as the reciprocal of the edge weight (*L_*ij*_* = 1/*w_*ij*_*). That is the higher of the edge weight, the shorter path length.
Small-worldness (σ)	$\sigma =\frac{\gamma }{\lambda }=\frac{C_{w}/C_{w}^{rand}}{L_{w}/L_{w}^{rand}}$	σ measures the small-world property. $C_{w}^{rand}$ and $L_{w}^{rand}$ represent the mean indices derived from 100 matched random networks. These random networks were derived from the original brain network by randomly rewiring the edges between nodes while preserving the degree distribution and connectedness.
Global efficiency (*E_*global*_*)	$E_{global}=\frac{1}{N\left(N-1\right)}\underset{i\neq j\in N}{\sum }\frac{1}{\min\left\{L_{ij}\right\}}$	*E_*global*_* measures the global efficiency of parallel information transfer in the network and it is inversely related to *L_*w*_*.
Local efficiency (*E_*local*_*)	$E_{local}=\frac{1}{N}\underset{i\in N}{\sum }E_{global}\left(i\right)$	*E_*local*_* measures the mean local efficiency of the network. *E_*global*_*(*i*) is the global efficiency of the subgraph of the neighbour of node *i*.
**REGIONAL PROPERTIES**		
Nodal efficiency (*E_*nodal*_*)	$E_{nodal}\left(i\right)=\frac{1}{N}\underset{i\neq j\in N}{\sum }\frac{1}{\min\left\{L_{ij}\right\}}$	*E_*nodal*_*(*i*) is the inverse of the harmonic mean of the shortest path length between node *i* and all other nodes. It measures the ability of information transmission of node *i* in the network: a node with high *E_*nodal*_* indicates great interconnectivity with other regions in the network.

Of note, each of the obtained hemispheric functional brain network was thresholded to a fixed sparsity value, but retaining the supra-threshold weights, prior to graph theory analysis to ensure that the wiring cost of each participant was comparable (Achard and Bullmore, 2007; He et al., 2009). In the current work, a wide range of sparsity (i.e., 0.1–0.35) with an interval of 1% was selected for graph theoretical analysis of hemispheric functional brain network to maintain the reachability of the network and allow prominent small-world properties. An integrated network metric was then estimated for all global and regional functional network metrics over the predefined sparsity range (Achard and Bullmore, 2007; He et al., 2009).

### Statistical analysis

#### Interhemispheric differences

Previous neuroimaging studies showed gender effect in structural and functional differences in brain asymmetry (Tian et al., 2011; Ingalhalikar et al., 2014; Sun et al., 2015). To detect whether there was significant hemispheric effect in any of the network metrics that were independent of the potential gender influence, a univariate analysis of covariance (ANCOVA) was performed separately on network measures of both functional and structural hemisphere networks with a threshold for significance of *p* < 0.05 (FDR-corrected). Gender was included as a covariate.

To determine the significance levels of lateralized connections, a network based statistical (NBS) analysis (Zalesky et al., 2010) was applied separately on the hemispheric structural and functional networks. Firstly, we performed a two-tailed paired *t*-test for each connection between both hemispheres and obtained *t* statistics for each edge. This step enables us to examine the maximal connected components (subnetworks) after setting a set of suprathreshold of the statistics. Subsequently, a non-parametric permutation test with 5,000 iterations was performed to obtain an empirical null distribution of the size of the maximal connected components and estimate the significance for each subnetwork. At each permutation, all of the hemispheric brain networks were randomly allocated into one of the two hemispheres. Next, the maximal connected component size was obtained using the same *t*-statistic threshold. Then the NBS-corrected *p*-value was determined through calculating the proportion of the 5,000 permutations where the maximal connected component was larger than that of the original grouping of left and right hemispheres. Detailed description about NBS method could be found in Zalesky et al. (2010).

#### Relationship between the network metrics and behavioral measures

In order to assess the relationship between the network metrics and behavioral measures, an index of asymmetry scores were calculated (Iturria-Medina et al., 2011; Sun et al., 2017): *AS*(*X*) = 100 × [*X*(*R*) – *X*(*L*)] / [*X*(*R*) + *X*(*L*)], where *X*(*R*) and *X*(*L*) stand for the network measures of the right and left hemispheres, respectively. The *AS*(*X*) index, ranging between +100 and −100, incorporated the relative network metrics over both hemispheres, which allow us to uncover the differences between the right and left hemispheres. Of note, for all network measures except *L*_*w*_, positive *AS*(*X*) indicates prominent rightward asymmetry and *AS*(*X*) is negative when metric *X* showed significant leftward predilection. Given that longer *L*_*w*_ suggests less efficient global integration, positive *AS*(*L*_*w*_) indicates a leftward advantage of global integration and negative *AS*(*L*_*w*_) represents rightward predilection.

Relationship between the hemispheric asymmetry scores and behavioral measures were also explored in the current work. Specifically, partial correlation was employed with the covariates of age, gender, handedness, and years of education. To limit the number of association calculations, only network metrics that displayed significant hemispheric effect were chosen for the analysis. The threshold value for establishment of a significant relationship was set at *p* < 0.05. Unless stated otherwise, all statistical analyses were performed using SPSS 17 software (IBM, Armonk, New York).

## Results

### Global properties of hemispheric networks

In line with previous findings (Iturria-Medina et al., 2011; Tian et al., 2011), we found prominent features of small-word topology in the hemispheric networks; that is, greater local clustering and comparable short path lengths relative to the random networks (data not shown), in both structural and functional hemispheric brain networks.

**Figure 2:**
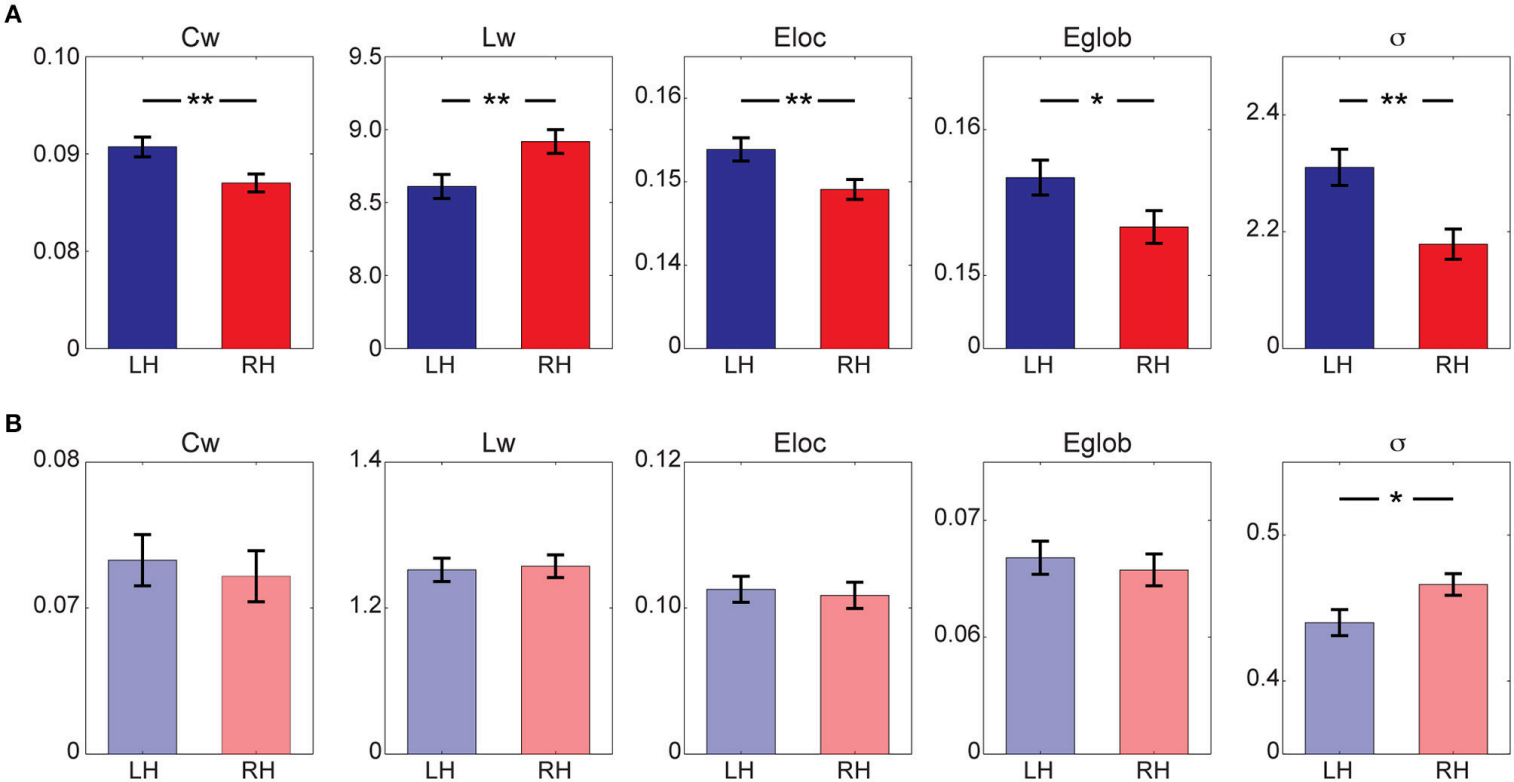
Global network properties for **(A)** structural hemispheric network and **(B)** functional hemispheric network. Bars represent mean ± standard error. ^*^Indicates *p* < 0.05; ^**^Indicates *p* < 0.01. LH, left hemisphere; RH, right hemisphere.

Quantitative statistical analysis revealed different lateralization patterns between structural and functional hemispheric networks (Figure 2). Particularly, a significant leftward predilection of local integration [Left > Right: *C*_*w*_, *F*_(1, 149)_ = 7.378, *p* = 0.007; *E*_*local*_, *F*_(1, 149)_ = 6.858, *p* = 0.010], together with a left hemispheric advantage in the global integration [Left < Right: *L*_*w*_, *F*_(1, 149)_ = 7.155, *p* = 0.008; Left > Right: *E*_*global*_, *t*_(77)_ = 4.275, *p* = 0.040] was observed in structural hemispheric networks, leading to a higher small-worldness in the left hemisphere [Left > Right: σ, *F*_(1, 149)_ = 10.598, *p* = 0.001]. In the functional brain networks, a significant hemispheric effect was observed in small-worldness [Left < Right: σ, *F*_(1, 149)_ = 5.080, *p* = 0.026], indicating a rightward predilection of optimal architecture in the right hemisphere. No significant hemispheric effects (*p* > 0.05) were observed for other metrics (*C*_*w*_, *L*_*w*_, *E*_*local*_, and *E*_*global*_) derived from the functional hemispheric networks.

### Regional properties of hemispheric networks

**Figure 3:**
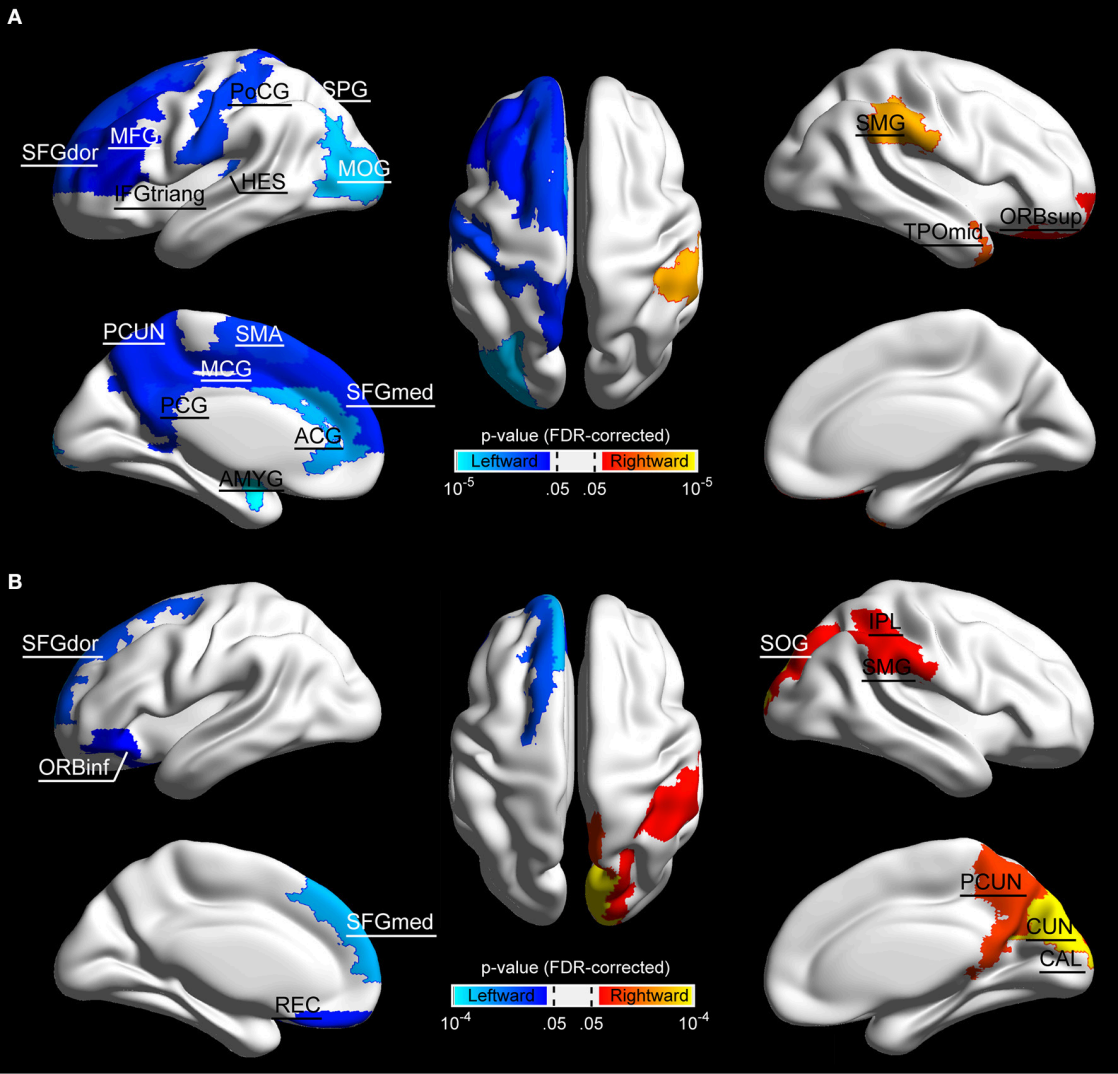
The surface distribution of cortical regions showing significant hemisphere effect in **(A)** structural network and **(B)** functional network. Color bar indicates *p*-values, and the threshold value for establishing significance was set *p* < 0.05 (FDR-corrected). Significant regions were overlaid on inflated surface maps with BrainNet Viewer software (Xia et al., 2013). For the abbreviations of the cortical regions, see Table 2.

We further localized the regions showing significant hemispheric effect. Specifically, significant hemispheric effect (*p* < 0.05, FDR-corrected) was revealed in 17 regions across the cerebral cortex in structural brain networks (Figure 3A). Among these brain regions, 14 regions (including the amygdala [AMYG], anterior cingulate gyrus [ACG], heschl gyrus [HES], inferior frontal gyrus, triangle part [IFGtriang], middle frontal gyrus [MFG], middle occipital gyrus [MOG], middle cingulate gyrus [MCG], postcentral gyrus [PoCG], posterior cingulate gyrus [PCG], precuneus [PCUN], superior frontal gyrus, medial part [SFGmed], superior frontal gyrus, dorsal part [SFGdor], supplementary motor area [SMA], and superior parietal gyrus [SPG]) mainly located in the inferior frontal and medial areas showed leftward lateralization of regional efficiency, whereas only three regions (including the supramarginal gyrus [SMG], temporal pole, middle part [TPOmid], orbitofrontal gyrus, superior part [ORBsup]), predominantly located temporal area, exhibited a rightward advantage in regional efficiency. In functional brain networks, 10 regions exhibited significant hemispheric effect (*p* < 0.05, FDR-corrected), where four regions (orbitofrontal gyrus, inferior part [ORBinf], gyrus rectus [REC], SFGdor, and superior frontal gyrus, medial part [SFGmed]) showed leftward advantage and the other six regions (including the calcarine fissure [CAL], cuneus [CUN], inferior parietal lobule [IPL], PCUN, SMG, and superior occipital gyrus [SOG]) mainly located in the parieto-occipital area showed a rightward predilection (Figure 3B).

### Lateralized inter-regional connectivity

**Figure 4:**
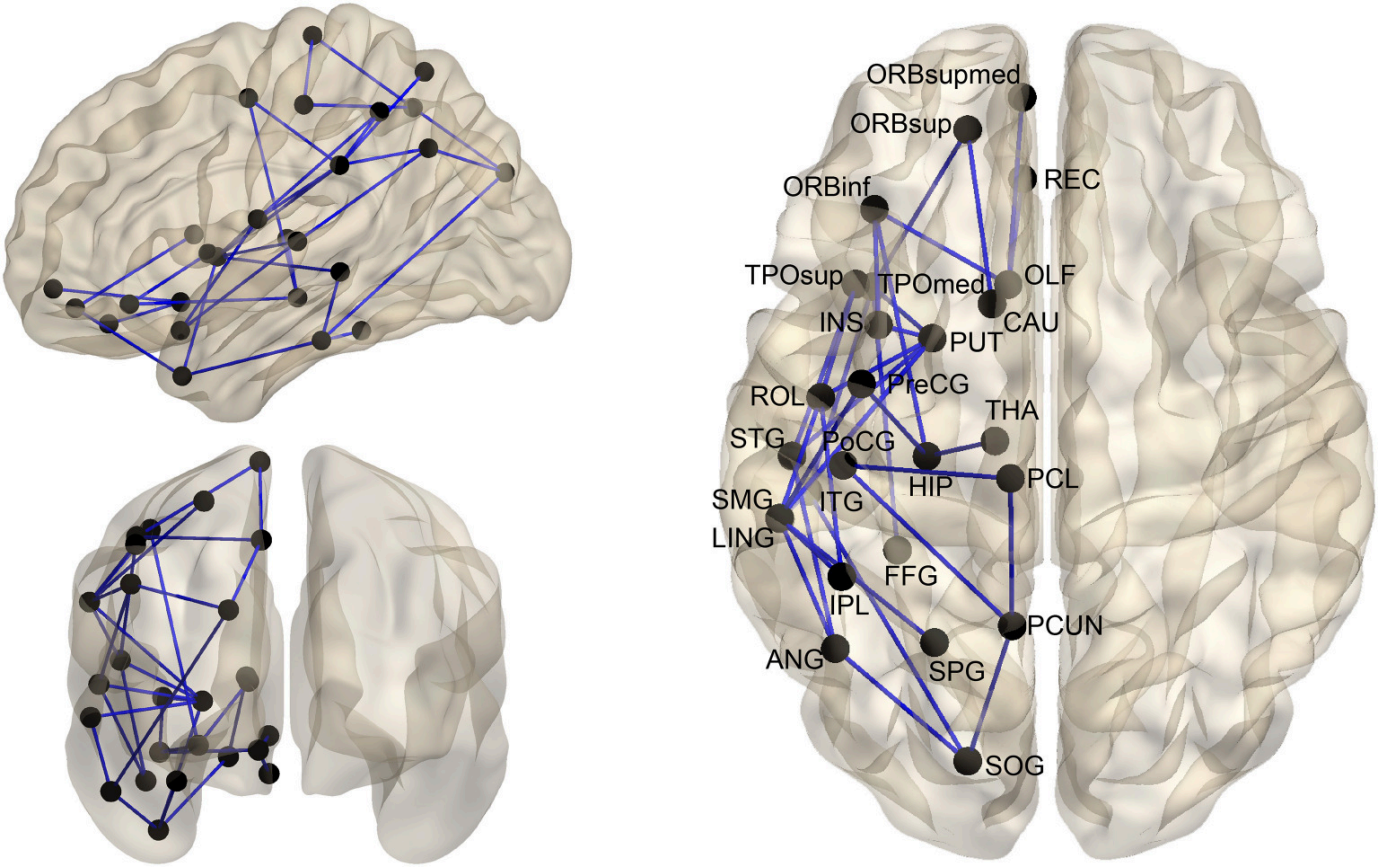
The distribution of structural connections showing significant (*p* < 0.05, NBS-corrected) hemisphere effect. These connections formed a single connected network with 26 nodes and 33 connections. For the abbreviations of the cortical regions, see Table 2.

We used NBS method to identify the significantly lateralized inter-regional connectivity between both hemispheres. Specifically, in structural hemispheric networks, a significant leftward predilection (*p* < 0.05, NBS-corrected) was revealed in a single connected network with 26 nodes and 33 edges (Figure 4). Visual inspection showed that the edges with significant hemispheric effect mainly connected brain regions between parieto-occipital and temporal/orbitofrontal areas. The involved nodal regions included the parieto-occipital (the PCUN, SPG, SOG, angular gyrus [ANG], IPL, paracentral lobule [PCL], SMG, ROL, lingual gyrus [LING], and PoCG), the temporal (the superior temporal gyrus [STG], temporal pole, superior part [TPOsup], TPOmid, ITG, and FFG), the orbitofrontal (the orbitofrontal gyrus, medial part [ORBmed], ORBsup, REC, olfactory [OLF], ORBinf, INS, and PreCG), and some subcortical areas (the CAU, putamen [PUT], THA, and HIP). No statistically significant (*p* < 0.05, NBS-corrected) lateralized connectivity was revealed in functional hemispheric networks.

### Relationship between hemispheric asymmetry and behavioral measures

Notably, given that the behavioral measures covered multiple domains and well-known localized process across different brain areas, these significant associations exhibited region-dependent patterns. In the global network metrics showing significant hemispheric predilection, a significant association (*r* = 0.275, *p* = 0.018) between the asymmetry scores of the weighted characteristic path (*AS*(*L*_*w*_)) in structural brain networks and scores of RAVLT_ir_ was revealed, whereas the asymmetry scores of local efficiency (*AS*(*E*_*local*_)) was found to be associated with the scores of SDMT_oral_ (*r* = −0.252, *p* = 0.031), Boston naming (*r* = −0.231, *p* = 0.049), and MoCA (*r* = −0.307, *p* = 0.008). No statistically significant (*p* > 0.05) association was revealed between behavioral measures and global network metric in functional brain network. For the regional asymmetry scores in structural brain network, 6 regions among 17 that showed significant hemispheric effect exhibited significant correlations (*p* < 0.05, uncorrected) with specific behavioral measures. Specifically, significantly negative relationship was revealed between the asymmetry scores of *E*_*nodal*_ of SFGdor and PCG and the Boston naming scores [*AS*(*E*_*nodal*_(SFGdor)), *r* = −0.271, *p* = 0.020; *AS*(*E*_*nodal*_(PCG)), *r* = −0.230, *p* = 0.050]; between *AS*(*E*_*nodal*_(MCG)) and SDMT_written_ scores (*r* = −0.248, *p* = 0.035); and between *AS*(*E*_*nodal*_(IFGtriang)) and the CTT1 scores (*r* = −0.260, *p* = 0.026). Moreover, significantly positive relationship was found between the asymmetry scores of *E*_*nodal*_ of HES and MoCA scores (*r* = 0.295, *p* = 0.011) as well as *AS*(*E*_*nodal*_(TPOmid)) and CTT2 (*r* = 0.268, *p* = 0.022). In functional brain network, the asymmetry scores of nodal efficiency of SFGdor were significantly correlated with the SDMT_written_ scores (*r* = 0.299, *p* = 0.010) and the asymmetry scores of nodal efficiency of SMG were positively correlated with block design scores (*r* = 0.273, *p* = 0.020).

## Discussion

In the current study, using multimodal neuroimaging techniques and graph theory analysis, we examined the hemispheric asymmetry in healthy aging adults. The significant findings are as follows: first, as expected, the optimal small-world properties were revealed in both structural and functional hemispheric networks; second, distinct hemispheric lateralization patterns were found between structural and functional brain networks at both global and local levels; third, the asymmetry scores of network metrics were correlated with the behavioral performance of cognitive function. These findings are discussed in greater detail below.

Recent advances of graph theoretical analysis and the identification of small-world architecture (high local clustering and short paths between brain regions) has significantly augmented our understanding about the topological organization of brain networks (Bullmore and Sporns, 2009; He and Evans, 2010; Sporns, 2011). Compared to serial or hierarchical processing, such small-world architecture represents an optimal network topology that keep a balance between local segregation and global integration (Watts and Strogatz, 1998). Specifically, high local clustering could facilitate specialized local cognitive function while short paths ensure efficient parallel information processing, therefore facilitating complex cognitive brain function (Sporns, 2011). In fact, convergent evidence has shown the presence of the small-world properties in healthy subjects at the whole-brain level (Bassett and Bullmore, 2009; Bullmore and Sporns, 2009; Sporns, 2011). Until quite recently, the optimal small-world characteristics were revealed in hemispheric brain networks (Iturria-Medina et al., 2011; Tian et al., 2011; Ratnarajah et al., 2013; Caeyenberghs and Leemans, 2014; Sun et al., 2017; Yang et al., 2017). Therefore, our observations of optimal small-world properties at hemispheric level extend these earlier findings and provided new multimodal neuroimaging evidence to demonstrate efficient information processing within each hemisphere similar to that of the whole brain.

In addition, distinct hemispheric lateralization patterns were observed in structural and functional brain networks. Specifically, a leftward advantage of network topology was revealed in the structural hemispheric networks. This finding was consistent with prior observations from structural network study of hemispheric asymmetry in old adults (Caeyenberghs and Leemans, 2014). Together with previous studies of structural connectivity asymmetry across different ages (Iturria-Medina et al., 2011; Ratnarajah et al., 2013; Caeyenberghs and Leemans, 2014; Zhong et al., 2016; Sun et al., 2017), we could clearly observe a developmental trend in topological asymmetry between hemispheric brain networks. For instance, a leftward predilection of network efficiency was firstly observed in neonatal brains that might result from the adaption to lateralized primary functional needs such as motor, language, and memory functions (Ratnarajah et al., 2013). From childhood to adulthood, the asymmetry undergoes a developmental progression as a result of bilateral cortical maturity, which leads to the right-larger-than-left asymmetry (Zhong et al., 2016). This rightward asymmetry might be attributed to broader cognitive process including visuospatial integration and attentional processing interact in the right hemisphere in comparison to more specialized cognitive process like language in the left hemisphere (Iturria-Medina et al., 2011). Such a right-larger-than-left asymmetry pattern was turned over in older adults, due to normal aging-related brain morphometric changes (Thambisetty et al., 2010; Lemaitre et al., 2012). Moreover, in functional brain networks, our observation of symmetric network topology between left and right hemispheres was in line with one recent study of hemispheric related differences in small-world brain networks (Tian et al., 2011). Given that subjects enrolled in Tian et al. (2011) were healthy young adults, our findings may therefore suggest a compensatory reaction of hemispheric functional brain networks to aging-related structural alterations (Cabeza, 2002; Dolcos et al., 2002; Ferreira and Busatto, 2013; Daselaar et al., 2015). In fact, accumulating evidences have suggested that a distributed processing was typically revealed in older adults in response to the demands of aging (Davis et al., 2012).

In comparison with the previous studies investigating hemispheric asymmetry at regional morphology level (e.g., cortical thickness, gray/white matter volume), regional asymmetry was assessed in terms of interconnectivity characteristics of each region between two hemispheres in this work. In line with the well-documented leftward asymmetry in language, motor and visual functions (Mesulam, 1998), regions with significant leftward asymmetry of nodal efficiency were revealed in the inferior frontal areas, precentral gyrus, postcentral gyrus, middle occipital gyrus, which was consistent with similar cortical thickness (Luders et al., 2006), morphometric (Good et al., 2001), and structural connectivity results (Caeyenberghs and Leemans, 2014; Sun et al., 2017). Moreover, we found regions with rightward predilection of *E*_*nodal*_ mainly located in the temporal areas, corresponding to the rightward predilection in memory functioning (Mesulam, 1998). More interestingly, we found a leftward dominance of regional efficiency asymmetry in structural brain networks (leftward/rightward = 14/3). Most of these regions with significant hemispheric asymmetry were identified as hubs (regions with higher interconnectivity, including the postcentral gyrus, superior frontal gyrus, middle frontal gyrus, precuneus, and middle occipital gyrus) in this study (data not shown) and in previous brain connectome studies (Wu et al., 2012; van den Heuvel and Sporns, 2013). The NBS analysis also revealed a significantly lateralized structural subnetwork in left hemisphere. Due to its higher interconnectivity, hub regions played a central role in receiving and integrating multiple inputs from different cortical regions. We therefore speculate that the profoundly asymmetric regions and connections may indicate more integrated network topology in left hemisphere, which led to our being able to reveal leftward predilection of network efficiency in structural network.

In functional hemispheric networks, however, a left-anterior-right-posterior asymmetric pattern was observed in regional efficiency. Particularly, consistent with structural findings, leftward advantage was mainly revealed in brain regions with well-known lateralized cognitive functions, such as language. In fact the superior frontal gyrus and inferior frontal gyrus have been repeatedly reported to be functionally asymmetric (Toga and Thompson, 2003). Moreover, our finding of rightward regional asymmetry in the posterior areas was in line with prior observations in functional (Liu et al., 2009) and structural (Iturria-Medina et al., 2011) network studies. According to Liu et al. (2009), these rightward asymmetries were attributed to right-lateralized visuospatial processing. Further inspection of hemispheric asymmetry pattern in functional networks, we found a symmetric pattern (leftward/rightward = 4/6) of regional efficiency asymmetry, which corroborated our observation of a symmetric global hemispheric topology.

Another interesting observation is that the asymmetry scores of network metrics were associated with behavioral measures. Particularly, significant associations were revealed between behavioral measures and asymmetry scores of global network metrics in structural networks. Given that *L*_*w*_ measures the overall routing efficiency of the network, the shorter *L*_*w*_, the higher global integration of the network. Therefore, the observed significant relationship suggested that the better behavioral performance was associated with more efficient network topology in the left hemisphere. According to Iturria-Medina, dedicated specialized networks were embedded in the left hemisphere to achieve its leading role for highly demanding specific process such as language and motor actions (Iturria-Medina et al., 2011). It is therefore not surprising to observe a strong association between leftward predilection of network efficiency and language-related behavioral measures (e.g., SDMT_oral_ and Boston naming). In terms of regional efficiency, we observed significant relationship between hemispheric lateralization of several well-documented language-related regions (e.g., triangular part of the inferior frontal gyrus) and behavioral test such as color trails test. This significant relationship at both global and regional levels revealed in structural brain networks was mostly absent in functional brain networks due to the symmetric topology. Taken together, our findings of complex relationship between network metrics and behavioral measures reiterated the distinct hemispheric asymmetry patterns between functional and structural brain networks. Likewise, a robust relationship between abnormal network topology and aging-related performance decline has emerged (Wang et al., 2013; Dai and He, 2014; Yang et al., 2017). Our exploratory findings therefore provide further support of using brain network properties as potential biomarkers for evaluation of the behavioral performance in healthy-aging population.

There are also several issues that need to be further addressed. First, the previously validated and widely used AAL template was employed here for the network construction to ensure the comparability needed for both imaging modalities as well as to maximize the number of existing studies with which our results could be directly compared without the need to determine a template-to-template mapping between discordant regional definitions (Sun et al., 2016b). Nonetheless, a potential confounding factor of different sizes of ROIs may influence the link weight among the network nodes (Wang et al., 2012). To address this issue, a streamline density approach was utilized to account for the different sizes of the ROIs (Buchanan et al., 2014). Given that there is as yet no widely-accepted means for defining network nodes for connectomic analyses (Fornito et al., 2013) and the best choice of edge weight definition to accurately represent the neurobiological connectivity is far from fully understood (Jones, 2010; Smith et al., 2011), we believe that new advances in brain parcellation approaches and edge weighting methods, examining the topological characteristics of hemispheric networks across the life scan are of importance for better understanding the hemispheric-specific developmental trend in the brain connectome. Second, a computationally inexpensive deterministic tractography method was employed to reconstruct the structural brain networks (Mori et al., 1999). However, this fiber tracking method may become hindered in correctly tracing fiber streamlines when the directional information at some point along the tract is not univocal (i.e., fiber crossing) (Jones et al., 2013). This may in turn result in an underrepresentation of the number of connections of the connectome. We assessed the credibility of our tracking results through inspecting and comparing several well-known WM fiber bundles with other studies (Gong et al., 2009; Li et al., 2009; Sun et al., 2017), and found comparable tracking results that were faithful to the human WM anatomy (data not shown). Although probabilistic tractography method with much higher sampling directions is advantageous in overcoming fiber crossing problem (Behrens et al., 2007), recent research has indicated that such method would yield dense connectomes with increased false positive connections and reduced specificity of connectome constructions (Zalesky et al., 2016). Nevertheless, future studies with cautious application of advanced probabilistic fiber tracking method and cross-fiber models to high-quality data is recommended to confirm our observations. Thirdly, using a cross-sectional design, Zhou et al. had investigated the cortical thickness asymmetry from childhood to older adulthood and showed that hemispheric asymmetry was increased during aging (Zhou et al., 2013). In line with this notion, a recent longitudinal study of cortical thickness in normal aging reported a general thinning in the left hemisphere together with a localized thinning in the right parietal regions (Thambisetty et al., 2010). Although this cross-section study shed some of the first light into quantitative investigation of hemispheric asymmetry in older adults, follow-up longitudinal brain connectome studies across the life span (Zuo et al., 2017) are needed to elucidate that how the hemispheric asymmetry in structural and functional brain networks are conserved or affected over time. Finally, an uncorrected *p*-value of 0.05 was employed for establishing the significance and presenting the correlation results. Although we mainly focused on the interpretation of the general pattern of the findings in the current work, the possibility that some of the results may have occurred by chance cannot be completely ruled out, therefore, some caution is needed when interpreting these results. The primary focus of the current work was to investigate the lateralized network topology, and the association analyses were exploratory in nature. We also provided the exact statistical analysis results for the readers' interpretation. Nonetheless, future studies using an independent study sample and hypothesis-driven study design are of interest.

In conclusion, using multimodal brain connectome, we investigate the hemispheric asymmetry in healthy aging adults. We found that although prominent small-world properties were preserved in both hemispheres, distinct hemispheric asymmetry patterns were observed between functional and structural brain networks at both local and global levels. These findings indicate that a complex brain network analysis could be a profitable tool for investigating individual differences in brain structure and function. Further work with a longitudinal design could be conducted to examine the progression of the hemispheric asymmetry as well as the complex structure-function relationships during normal aging.

## Author contributions

YS, JS, and LF conceptualized the study. LF collected the data. YS and JL analyzed the data. YS interpreted the results and wrote the paper. All authors contributed and approved the final manuscript for publication.

### Conflict of interest statement

The authors declare that the research was conducted in the absence of any commercial or financial relationships that could be construed as a potential conflict of interest.

## References

[B1] Achard S. Bullmore E. (2007). Efficiency and cost of economical brain functional networks. PLoS Comput. Biol. 3:e17. 10.1371/journal.pcbi.0030017 17274684 PMC1794324

[B2] Anderson J. S. Druzgal T. J. Lopez-Larson M. Jeong E. K. Desai K. Yurgelun-Todd D. (2011). Network anticorrelations, global regression, and phase-shifted soft tissue correction. Hum. Brain Mapp. 32, 919–934. 10.1002/hbm.21079 20533557 PMC3220164

[B3] Ashburner J. (2007). A fast diffeomorphic image registration algorithm. Neuroimage 38, 95–113. 10.1016/j.neuroimage.2007.07.007 17761438

[B4] Bassett D. S. Bullmore E. T. (2009). Human brain networks in health and disease. Curr. Opin. Neurol. 22, 340–347. 10.1097/WCO.0b013e32832d93dd 19494774 PMC2902726

[B5] Behrens T. E. Berg H. J. Jbabdi S. Rushworth M. F. Woolrich M. W. (2007). Probabilistic diffusion tractography with multiple fibre orientations: what can we gain? Neuroimage 34, 144–155. 10.1016/j.neuroimage.2006.09.018 17070705 PMC7116582

[B6] Boccaletti S. Latora V. Moreno Y. Chavez M. Hwang D. U. (2006). Complex networks: structure and dynamics. Phys. Rep. 424, 175–308. 10.1016/j.physrep.2005.10.009

[B7] Buchanan C. R. Pernet C. R. Gorgolewski K. J. Storkey A. J. Bastin M. E. (2014). Test-retest reliability of structural brain networks from diffusion MRI. Neuroimage 86, 231–243. 10.1016/j.neuroimage.2013.09.054 24096127

[B8] Bullmore E. Sporns O. (2009). Complex brain networks: graph theoretical analysis of structural and functional systems. Nat. Rev. Neurosci. 10, 186–198. 10.1038/nrn2575 19190637

[B9] Cabeza R. (2002). Hemispheric asymmetry reduction in older adults: the HAROLD model. Psychol. Aging 17, 85–100. 10.1037/0882-7974.17.1.85 11931290

[B10] Caeyenberghs K. Leemans A. (2014). Hemispheric lateralization of topological organization in structural brain networks. Hum. Brain Mapp. 35, 4944–4957. 10.1002/hbm.22524 24706582 PMC6869817

[B11] Cao Y. Whalen S. Huang J. Berger K. L. DeLano M. C. (2003). Asymmetry of subinsular anisotropy by *in vivo* diffusion tensor imaging. Hum. Brain Mapp. 20, 82–90. 10.1002/hbm.10130 14505334 PMC3595070

[B12] Casey B. J. Galvan A. Hare T. A. (2005). Changes in cerebral functional organization during cognitive development. Curr. Opin. Neurobiol. 15, 239–244. 10.1016/j.conb.2005.03.012 15831409

[B13] Chang C. Glover G. H. (2009). Effects of model-based physiological noise correction on default mode network anti-correlations and correlations. Neuroimage 47, 1448–1459. 10.1016/j.neuroimage.2009.05.012 19446646 PMC2995588

[B14] Colcombe S. J. Kramer A. F. Erickson K. I. Scalf P. (2005). The implications of cortical recruitment and brain morphology for individual differences in inhibitory function in aging humans. Psychol. Aging 20, 363–375. 10.1037/0882-7974.20.3.363 16248697

[B15] Crow T. J. Ball J. Bloom S. R. Brown R. Bruton C. J. Colter N.. (1989). Schizophrenia as an anomaly of development of cerebral asymmetry. A postmortem study and a proposal concerning the genetic basis of the disease. Arch. Gen. Psychiatry 46, 1145–1150. 10.1001/archpsyc.1989.01810120087013 2589928

[B16] Cui Z. Zhong S. Xu P. He Y. Gong G. (2013). PANDA: a pipeline toolbox for analyzing brain diffusion images. Front. Hum. Neurosci. 7:42. 10.3389/fnhum.2013.00042 23439846 PMC3578208

[B17] Dai Z. He Y. (2014). Disrupted structural and functional brain connectomes in mild cognitive impairment and Alzheimer's disease. Neurosci. Bull. 30, 217–232. 10.1007/s12264-013-1421-0 24733652 PMC5562665

[B18] Daselaar S. M. Iyengar V. Davis S. W. Eklund K. Hayes S. M. Cabeza R. E. (2015). Less wiring, more firing: low-performing older adults compensate for impaired white matter with greater neural activity. Cereb. Cortex 25:983–990. 10.1093/cercor/bht289 24152545 PMC4366614

[B19] Davis S. W. Kragel J. E. Madden D. J. Cabeza R. (2012). The architecture of cross-hemispheric communication in the aging brain: linking behavior to functional and structural connectivity. Cereb Cortex 22, 232–242. 10.1093/cercor/bhr123 21653286 PMC3236798

[B20] Dolcos F. Rice H. J. Cabeza R. (2002). Hemispheric asymmetry and aging: right hemisphere decline or asymmetry reduction. Neurosci. Biobehav. Rev. 26, 819–825. 10.1016/S0149-7634(02)00068-4 12470693

[B21] Feng L. (2017). Ageing in a Community Environment Study (ACES) Cohort. Singapore: Encyclopedia of Geropsychology.

[B22] Feng L. Chong M. S. Lim W. S. Ng T. P. (2012). The Modified Mini-Mental State Examination test: normative data for Singapore Chinese older adults and its performance in detecting early cognitive impairment. Singapore Med. J. 53, 458–462. 22815014

[B23] Ferreira L. K. Busatto G. F. (2013). Resting-state functional connectivity in normal brain aging. Neurosci. Biobehav. Rev. 37, 384–400. 10.1016/j.neubiorev.2013.01.017 23333262

[B24] Folstein M. F. Folstein S. E. McHugh P. R. (1975). “Mini-mental state”. A practical method for grading the cognitive state of patients for the clinician. J. Psychiatr. Res. 12, 189–198. 10.1016/0022-3956(75)90026-6 1202204

[B25] Fornito A. Zalesky A. Breakspear M. (2013). Graph analysis of the human connectome: promise, progress, and pitfalls. Neuroimage 80, 426–444. 10.1016/j.neuroimage.2013.04.087 23643999

[B26] Galaburda A. M. LeMay M. Kemper T. L. Geschwind N. (1978). Right-left asymmetrics in the brain. Science 199, 852–856. 10.1126/science.341314 341314

[B27] Geschwind N. Levitsky W. (1968). Human brain: left-right asymmetries in temporal speech region. Science 161, 186–187. 10.1126/science.161.3837.186 5657070

[B28] Giedd J. N. Blumenthal J. Jeffries N. O. Castellanos F. X. Liu H. Zijdenbos A.. (1999). Brain development during childhood and adolescence: a longitudinal MRI study. Nat. Neurosci. 2, 861–863. 10.1038/13158 10491603

[B29] Giedd J. N. Snell J. W. Lange N. Rajapakse J. C. Casey B. J. Kozuch P. L.. (1996). Quantitative magnetic resonance imaging of human brain development: ages 4-18. Cereb. Cortex 6, 551–560. 10.1093/cercor/6.4.551 8670681

[B30] Gong G. Rosa-Neto P. Carbonell F. Chen Z. J. He Y. Evans A. C. (2009). Age- and gender-related differences in the cortical anatomical network. J. Neurosci. 29, 15684–15693. 10.1523/JNEUROSCI.2308-09.2009 20016083 PMC2831804

[B31] Good C. D. Johnsrude I. Ashburner J. Henson R. N. Friston K. J. Frackowiak R. S. (2001). Cerebral asymmetry and the effects of sex and handedness on brain structure: a voxel-based morphometric analysis of 465 normal adult human brains. Neuroimage 14, 685–700. 10.1006/nimg.2001.0857 11506541

[B32] Hausmann M. Gunturkun O. (2000). Steroid fluctuations modify functional cerebral asymmetries: the hypothesis of progesterone-mediated interhemispheric decoupling. Neuropsychologia 38, 1362–1374. 10.1016/S0028-3932(00)00045-2 10869579

[B33] Hausmann M. Gunturkun O. Corballis M. (2003). Age-related changes in hemispheric asymmetry depend on sex. Laterality 8, 277–290. 10.1080/13576500244000201 15513227

[B34] He Y. Evans A. (2010). Graph theoretical modeling of brain connectivity. Curr. Opin. Neurol. 23, 341–350. 10.1097/WCO.0b013e32833aa567 20581686

[B35] He Y. Wang J. Wang L. Chen Z. J. Yan C. Yang H.. (2009). Uncovering intrinsic modular organization of spontaneous brain activity in humans. PLoS ONE 4:e5226. 10.1371/journal.pone.0005226 19381298 PMC2668183

[B36] Herve P. Y. Zago L. Petit L. Mazoyer B. Tzourio-Mazoyer N. (2013). Revisiting human hemispheric specialization with neuroimaging. Trends Cogn. Sci. 17, 69–80. 10.1016/j.tics.2012.12.004 23317751

[B37] Ingalhalikar M. Smith A. Parker D. Satterthwaite T. D. Elliott M. A. Ruparel K.. (2014). Sex differences in the structural connectome of the human brain. Proc. Natl. Acad. Sci. U.S.A. 111, 823–828. 10.1073/pnas.1316909110 24297904 PMC3896179

[B38] Iturria-Medina Y. Perez Fernandez A. Morris D. M. Canales-Rodriguez E. J. Haroon H. A. Garcia Penton L.. (2011). Brain hemispheric structural efficiency and interconnectivity rightward asymmetry in human and nonhuman primates. Cereb. Cortex 21, 56–67. 10.1093/cercor/bhq058 20382642

[B39] Jahanshad N. Rajagopalan P. Hua X. Hibar D. P. Nir T. M. Toga A. W.. (2013). Genome-wide scan of healthy human connectome discovers SPON1 gene variant influencing dementia severity. Proc. Natl. Acad. Sci. U.S.A. 110, 4768–4773. 10.1073/pnas.1216206110 23471985 PMC3606977

[B40] Jenkinson M. Bannister P. Brady M. Smith S. (2002). Improved optimization for the robust and accurate linear registration and motion correction of brain images. Neuroimage 17, 825–841. 10.1006/nimg.2002.1132 12377157

[B41] Jones D. K. (2010). Challenges and limitations of quantifying brain connectivity *in vivo* with diffusion MRI. Imaging Med. 2, 341–355. 10.2217/iim.10.21

[B42] Jones D. K. Knosche T. R. Turner R. (2013). White matter integrity, fiber count, and other fallacies: the do's and don'ts of diffusion MRI. Neuroimage 73, 239–254. 10.1016/j.neuroimage.2012.06.081 22846632

[B43] Kim J. H. Lee J. W. Kim G. H. Roh J. H. Kim M. J. Seo S. W.. (2012). Cortical asymmetries in normal, mild cognitive impairment, and Alzheimer's disease. Neurobiol. Aging 33, 1959–1966. 10.1016/j.neurobiolaging.2011.06.026 21907459

[B44] Lancaster J. L. Kochunov P. V. Thompson P. M. Toga A. W. Fox P. T. (2003). Asymmetry of the brain surface from deformation field analysis. Hum. Brain Mapp. 19, 79–89. 10.1002/hbm.10105 12768532 PMC6872049

[B45] Latora V. Marchiori M. (2001). Efficient behavior of small-world networks. Phys. Rev. Lett. 87:198701. 10.1103/PhysRevLett.87.198701 11690461

[B46] Lemaitre H. Goldman A. L. Sambataro F. Verchinski B. A. Meyer-Lindenberg A. Weinberger D. R.. (2012). Normal age-related brain morphometric changes: nonuniformity across cortical thickness, surface area and gray matter volume? Neurobiol. Aging 33, 617 e1–617 e9. 10.1016/j.neurobiolaging.2010.07.013 20739099 PMC3026893

[B47] Li Y. H. Liu Y. Li J. Qin W. Li K. C. Yu C. S.. (2009). Brain anatomical network and intelligence. PLoS Comput. Biol. 5:e1000395. 10.1371/journal.pcbi.1000395 19492086 PMC2683575

[B48] Liew T. M. Feng L. Gao Q. Ng T. P. Yap P. (2015). Diagnostic utility of montreal cognitive assessment in the fifth edition of diagnostic and statistical manual of mental disorders: major and mild neurocognitive disorders. J. Am. Med. Dir. Assoc. 16, 144–148. 10.1016/j.jamda.2014.07.021 25282632

[B49] Liu H. Stufflebeam S. M. Sepulcre J. Hedden T. Buckner R. L. (2009). Evidence from intrinsic activity that asymmetry of the human brain is controlled by multiple factors. Proc. Natl. Acad. Sci. U.S.A. 106, 20499–20503. 10.1073/pnas.0908073106 19918055 PMC2777963

[B50] Lowe M. J. Mock B. J. Sorenson J. A. (1998). Functional connectivity in single and multislice echoplanar imaging using resting-state fluctuations. Neuroimage 7, 119–132. 10.1006/nimg.1997.0315 9558644

[B51] Luders E. Narr K. L. Thompson P. M. Rex D. E. Jancke L. Toga A. W. (2006). Hemispheric asymmetries in cortical thickness. Cereb. Cortex 16, 1232–1238. 10.1093/cercor/bhj064 16267139

[B52] Mesulam M. M. (1998). From sensation to cognition. Brain 121 (Pt 6), 1013–1052. 10.1093/brain/121.6.1013 9648540

[B53] Mori S. Crain B. J. Chacko V. P. van Zijl P. C. (1999). Three-dimensional tracking of axonal projections in the brain by magnetic resonance imaging. Ann. Neurol. 45, 265–269. 10.1002/1531-8249(199902)45:2<265::AID-ANA21>3.0.CO;2-3 9989633

[B54] Nasreddine Z. S. Phillips N. A. Bedirian V. Charbonneau S. Whitehead V. Collin I.. (2005). The Montreal Cognitive Assessment, MoCA: a brief screening tool for mild cognitive impairment. J. Am. Geriatr. Soc. 53, 695–699. 10.1111/j.1532-5415.2005.53221.x 15817019

[B55] Power J. D. Barnes K. A. Snyder A. Z. Schlaggar B. L. Petersen S. E. (2012). Spurious but systematic correlations in functional connectivity MRI networks arise from subject motion. Neuroimage 59, 2142–2154. 10.1016/j.neuroimage.2011.10.018 22019881 PMC3254728

[B56] Ratnarajah N. Rifkin-Graboi A. Fortier M. V. Chong Y. S. Kwek K. Saw S. M.. (2013). Structural connectivity asymmetry in the neonatal brain. Neuroimage 75, 187–194. 10.1016/j.neuroimage.2013.02.052 23501049 PMC3959921

[B57] Reiss A. L. Abrams M. T. Singer H. S. Ross J. L. Denckla M. B. (1996). Brain development, gender and IQ in children. A volumetric imaging study. Brain 119 (Pt 5), 1763–1774. 10.1093/brain/119.5.1763 8931596

[B58] Rubinov M. Sporns O. (2010). Complex network measures of brain connectivity: uses and interpretations. Neuroimage 52, 1059–1069. 10.1016/j.neuroimage.2009.10.003 19819337

[B59] Schachter S. C. Ransil B. J. Geschwind N. (1987). Associations of handedness with hair color and learning disabilities. Neuropsychologia 25, 269–276. 10.1016/0028-3932(87)90137-0 3574663

[B60] Schmidt M. (1996). Rey Auditory Verbal Learning Test: A Handbook. Los Angeles, CA: Western Psychological Services.

[B61] Shu N. Li X. Ma C. Zhang J. Chen K. Liang Y.. (2015). Effects of APOE promoter polymorphism on the topological organization of brain structural connectome in nondemented elderly. Hum. Brain Mapp. 36, 4847–4858. 10.1002/hbm.22954 26314833 PMC4715757

[B62] Smith S. M. Jenkinson M. Woolrich M. W. Beckmann C. F. Behrens T. E. Johansen-Berg H.. (2004). Advances in functional and structural MR image analysis and implementation as FSL. Neuroimage 23(Suppl. 1), S208–S219. 10.1016/j.neuroimage.2004.07.051 15501092

[B63] Smith S. M. Miller K. L. Salimi-Khorshidi G. Webster M. Beckmann C. F. Nichols T. E.. (2011). Network modelling methods for FMRI. Neuroimage 54, 875–891. 10.1016/j.neuroimage.2010.08.063 20817103

[B64] Song J. W. Mitchell P. D. Kolasinski J. Ellen Grant P. Galaburda A. M. Takahashi E. (2014). Asymmetry of white matter pathways in developing human brains. Cereb. Cortex 25, 2883–2893. 10.1093/cercor/bhu084 24812082 PMC4537435

[B65] Song X. W. Dong Z. Y. Long X. Y. Li S. F. Zuo X. N. Zhu C. Z.. (2011). REST: a toolkit for resting-state functional magnetic resonance imaging data processing. PLoS ONE 6:e25031. 10.1371/journal.pone.0025031 21949842 PMC3176805

[B66] Sporns O. (2011). The human connectome: a complex network. Ann. N. Y. Acad. Sci. 1224, 109–125. 10.1111/j.1749-6632.2010.05888.x 21251014

[B67] Sun Y. Chen Y. Collinson S. L. Bezerianos A. Sim K. (2017). Reduced hemispheric asymmetry of brain anatomical networks is linked to schizophrenia: a connectome study. Cereb. Cortex 27, 602–615. 10.1093/cercor/bhv25 26503264

[B68] Sun Y. Chen Y. Lee R. Bezerianos A. Collinson S. L. Sim K. (2016a). Disruption of brain anatomical networks in schizophrenia: a longitudinal, diffusion tensor imaging based study. Schizophr. Res. 171, 149–157. 10.1016/j.schres.2016.01.025 26811255

[B69] Sun Y. Dai Z. Li J. Collinson S. L. Sim K. (2016b). Modular-level alterations of structure-function coupling in schizophrenia connectome. Hum. Brain Mapp. 38, 2008–2025. 10.1002/hbm.23501 28032370 PMC6867028

[B70] Sun Y. Lee R. Chen Y. Collinson S. Thakor N. Bezerianos A.. (2015). Progressive gender differences of structural brain networks in healthy adults: a longitudinal, diffusion tensor imaging study. PLoS ONE 10:e0118857. 10.1371/journal.pone.0118857 25742013 PMC4350987

[B71] Takao H. Abe O. Yamasue H. Aoki S. Sasaki H. Kasai K.. (2011a). Gray and white matter asymmetries in healthy individuals aged 21-29 years: a voxel-based morphometry and diffusion tensor imaging study. Hum. Brain Mapp. 32, 1762–1773. 10.1002/hbm.21145 20886579 PMC6870485

[B72] Takao H. Hayashi N. Ohtomo K. (2011b). White matter asymmetry in healthy individuals: a diffusion tensor imaging study using tract-based spatial statistics. Neuroscience 193, 291–299. 10.1016/j.neuroscience.2011.07.041 21824507

[B73] Thambisetty M. Wan J. Carass A. An Y. Prince J. L. Resnick S. M. (2010). Longitudinal changes in cortical thickness associated with normal aging. Neuroimage 52, 1215–1223. 10.1016/j.neuroimage.2010.04.258 20441796 PMC2910226

[B74] Thiebaut de Schotten M. Dell'Acqua F. Forkel S. J. Simmons A. Vergani F. Murphy D. G.. (2011a). A lateralized brain network for visuospatial attention. Nat. Neurosci. 14, 1245–1246. 10.1038/nn.2905 21926985

[B75] Thiebaut de Schotten M. Ffytche D. H. Bizzi A. Dell'Acqua F. Allin M. Walshe M.. (2011b). Atlasing location, asymmetry and inter-subject variability of white matter tracts in the human brain with MR diffusion tractography. Neuroimage 54, 49–59. 10.1016/j.neuroimage.2010.07.055 20682348

[B76] Thompson S. A. Patterson K. Hodges J. R. (2003). Left/right asymmetry of atrophy in semantic dementia: behavioral-cognitive implications. Neurology 61, 1196–1203. 10.1212/01.WNL.0000091868.28557.B8 14610120

[B77] Tian L. Wang J. Yan C. He Y. (2011). Hemisphere- and gender-related differences in small-world brain networks: a resting-state functional MRI study. Neuroimage 54, 191–202. 10.1016/j.neuroimage.2010.07.066 20688177

[B78] Toga A. W. Thompson P. M. (2003). Mapping brain asymmetry. Nat. Rev. Neurosci, 4, 37–48. 10.1038/nrn1009 12511860

[B79] Tzourio-Mazoyer N. Landeau B. Papathanassiou D. Crivello F. Etard O. Delcroix N.. (2002). Automated anatomical labeling of activations in SPM using a macroscopic anatomical parcellation of the MNI MRI single-subject brain. Neuroimage 15, 273–289. 10.1006/nimg.2001.0978 11771995

[B80] van den Heuvel M. P. Sporns O. (2013). Network hubs in the human brain. Trends Cogn. Sci. 17, 683–696. 10.1016/j.tics.2013.09.012 24231140

[B81] Van Dijk K. R. Sabuncu M. R. Buckner R. L. (2012). The influence of head motion on intrinsic functional connectivity MRI. Neuroimage 59, 431–438. 10.1016/j.neuroimage.2011.07.044 21810475 PMC3683830

[B82] Wachinger C. Salat D. H. Weiner M. Reuter M. (2016). Whole-brain analysis reveals increased neuroanatomical asymmetries in dementia for hippocampus and amygdala. Brain 139, 3253–3266. 10.1093/brain/aww243 27913407 PMC5840883

[B83] Wang J. Zuo X. Dai Z. Xia M. Zhao Z. Zhao X.. (2013). Disrupted functional brain connectome in individuals at risk for Alzheimer's disease. Biol. Psychiatry 73, 472–481. 10.1016/j.biopsych.2012.03.026 22537793

[B84] Wang Q. Su T. P. Zhou Y. Chou K. H. Chen I. Y. Jiang T.. (2012). Anatomical insights into disrupted small-world networks in schizophrenia. Neuroimage 59, 1085–1093. 10.1016/j.neuroimage.2011.09.035 21963918

[B85] Wang R. Benner T. Sorensen A. Wedeen V. (2007). Diffusion toolkit: a software package for diffusion imaging data processing and tractography. Proc. Intl. Soc. Mag. Reson. Med. 15:3720.

[B86] Watts D. J. Strogatz S. H. (1998). Collective dynamics of ‘small-world’ networks. Nature 393, 440–442. 10.1038/30918 9623998

[B87] Wu K. Taki Y. Sato K. Kinomura S. Goto R. Okada K.. (2012). Age-related changes in topological organization of structural brain networks in healthy individuals. Hum. Brain Mapp. 33, 552–568. 10.1002/hbm.21232 21391279 PMC6870030

[B88] Xia M. Wang J. He Y. (2013). BrainNet Viewer: a network visualization tool for human brain connectomics. PLoS ONE 8:e68910. 10.1371/journal.pone.0068910 23861951 PMC3701683

[B89] Yan C. G. Zang Y. F. (2010). DPARSF: a MATLAB toolbox for “Pipeline” data analysis of resting-state fMRI. Front. Syst. Neurosci. 4:13 10.3389/fnsys.2010.00013 20577591 PMC2889691

[B90] Yan C. G. Cheung B. Kelly C. Colcombe S. Craddock R. C. Di Martino A.. (2013). A comprehensive assessment of regional variation in the impact of head micromovements on functional connectomics. Neuroimage 76, 183–201. 10.1016/j.neuroimage.2013.03.004 23499792 PMC3896129

[B91] Yang C. Zhong S. Zhou X. Wei L. Wang L. Nie S. (2017). The abnormality of topological asymmetry between hemispheric brain white matter networks in alzheimer's disease and mild cognitive impairment. Front. Aging Neurosci. 9:261. 10.3389/fnagi.2017.00261 28824422 PMC5545578

[B92] Zalesky A. Fornito A. Bullmore E. T. (2010). Network-based statistic: identifying differences in brain networks. Neuroimage 53, 1197–1207. 10.1016/j.neuroimage.2010.06.041 20600983

[B93] Zalesky A. Fornito A. Cocchi L. Gollo L. L. van den Heuvel M. P. Breakspear M. (2016). Connectome sensitivity or specificity: which is more important? Neuroimage 142, 407–420. 10.1016/j.neuroimage.2016.06.035 27364472

[B94] Zhong S. He Y. Shu H. Gong G. (2016). Developmental changes in topological asymmetry between hemispheric brain white matter networks from adolescence to young adulthood. Cereb. Cortex 27, 2560–2570. 10.1093/cercor/bhw109 27114178

[B95] Zhou D. Lebel C. Evans A. Beaulieu C. (2013). Cortical thickness asymmetry from childhood to older adulthood. Neuroimage 83, 66–74. 10.1016/j.neuroimage.2013.06.073 23827331

[B96] Zuo X. N. He Y. Betzel R. F. Colcombe S. Sporns O. Milham M. P. (2017). Human Connectomics across the Life Span. Trends Cogn. Sci. 21, 32–45. 10.1016/j.tics.2016.10.005 27865786

[B97] Zuo X. N. Kelly C. Di Martino A. Mennes M. Margulies D. S. Bangaru S.. (2010). Growing together and growing apart: regional and sex differences in the lifespan developmental trajectories of functional homotopy. J. Neurosci. 30, 15034–15043. 10.1523/JNEUROSCI.2612-10.2010 21068309 PMC2997358

